# Investigating the Biological Relevance of *In Vitro*-Identified Putative Packaging Signals at the 5′ Terminus of Satellite Tobacco Necrosis Virus 1 Genomic RNA

**DOI:** 10.1128/JVI.02106-18

**Published:** 2019-04-17

**Authors:** Ioly Kotta-Loizou, Hadrien Peyret, Keith Saunders, Robert H. A. Coutts, George P. Lomonossoff

**Affiliations:** a Department of Life Sciences, Imperial College London, London, United Kingdom; b Department of Biological Chemistry, John Innes Centre, Norwich, United Kingdom; c Department of Biological and Environmental Sciences, University of Hertfordshire, Hatfield, United Kingdom; University of Maryland, College Park

**Keywords:** *Nicotiana benthamiana*, STNV-1, TNV-A, agroinfiltration, encapsidation, infection, packaging signals

## Abstract

Satellite tobacco necrosis virus 1 (STNV-1) is a model system for *in vitro* RNA encapsidation studies (N. Patel, E. C. Dykeman, R. H. A. Coutts, G. P. Lomonossoff, et al., Proc Natl Acad Sci U S A 112:2227–2232, 2015, https://doi.org/10.1073/pnas.1420812112; N. Patel, E. Wroblewski, G. Leonov, S. E. V. Phillips, et al., Proc Natl Acad Sci U S A 114:12255–12260, 2017, https://doi.org/10.1073/pnas.1706951114), leading to the identification of degenerate packaging signals (PSs) proposed to be involved in the recognition of its genome by the capsid protein (CP). The aim of the present work was to investigate whether these putative PSs can confer selective packaging of STNV-1 RNA *in vivo* and to assess the prospects of using decoy RNAs in antiviral therapy. We have developed an *in planta* packaging assay based on the transient expression of STNV-1 CP and have assessed the ability of the resulting virus-like particles (VLPs) to encapsidate mutant STNV-1 RNAs expected to have different encapsidation potential based on *in vitro* studies. The results revealed that >90% of the encapsidated RNAs are host derived, although there is some selectivity of packaging for STNV-1 RNA and certain host RNAs. Comparison of the packaging efficiencies of mutant STNV-1 RNAs showed that they are encapsidated mainly according to their abundance within the cells, rather than the presence or absence of the putative PSs previously identified from *in vitro* studies. In contrast, subsequent infection experiments demonstrated that host RNAs represent only <1% of virion content. Although selective encapsidation of certain host RNAs was noted, no direct correlation could be made between this preference and the presence of potential PSs in the host RNA sequences. Overall, the data illustrate that the differences in RNA packaging efficiency identified through *in vitro* studies are insufficient to explain the specific packaging of STNV-1 RNA.

**IMPORTANCE** Viruses preferentially encapsidate their own genomic RNA, sometimes as a result of the presence of clearly defined packaging signals (PSs) in their genome sequence. Recently, a novel form of short degenerate PSs has been proposed (N. Patel, E. C. Dykeman, R. H. A. Coutts, G. P. Lomonossoff, et al., Proc Natl Acad Sci U S A 112:2227–2232, 2015, https://doi.org/10.1073/pnas.1420812112; N. Patel, E. Wroblewski, G. Leonov, S. E. V. Phillips, et al., Proc Natl Acad Sci U S A 114:12255–12260, 2017, https://doi.org/10.1073/pnas.1706951114) using satellite tobacco necrosis virus 1 (STNV-1) as a model system for *in vitro* studies. It has been suggested that competing with these putative PSs may constitute a novel therapeutic approach against pathogenic single-stranded RNA viruses. Our work demonstrates that the previously identified PSs have no discernible significance for the selective packaging of STNV-1 *in vivo* in the presence and absence of competition or replication: viral sequences are encapsidated mostly on the basis of their abundance within the cell, while encapsidation of host RNAs also occurs. Nevertheless, the putative PSs identified in STNV-1 RNA may still have applications in bionanotechnology, such as the *in vitro* selective packaging of RNA molecules.

## Abstract (precis)

Viruses preferentially encapsidate their own genomic RNA, sometimes as a result of the presence of clearly defined packaging signals (PSs) in their genome sequence. Recently, a novel form of short degenerate PSs has been proposed (N. Patel, E. C. Dykeman, R. H. A. Coutts, G. P. Lomonossoff, et al., Proc Natl Acad Sci U S A 112:2227–2232, 2015, https://doi.org/10.1073/pnas.1420812112; N. Patel, E. Wroblewski, G. Leonov, S. E. V. Phillips, et al., Proc Natl Acad Sci U S A 114:12255–12260, 2017, https://doi.org/10.1073/pnas.1706951114) using satellite tobacco necrosis virus 1 (STNV-1) as a model system for *in vitro* studies. It has been suggested that competing with these putative PSs may constitute a novel therapeutic approach against pathogenic single-stranded RNA viruses. Our work demonstrates that the previously identified PSs have no discernible significance for the selective packaging of STNV-1 *in vivo* in the presence and absence of competition or replication: viral sequences are encapsidated mostly on the basis of their abundance within the cell, while encapsidation of host RNAs also occurs. Nevertheless, the putative PSs identified in STNV-1 RNA may still have applications in bionanotechnology, such as the *in vitro* selective packaging of RNA molecules.

## INTRODUCTION

Satellite tobacco necrosis virus 1 (STNV-1) is a satellite virus that requires the presence of tobacco necrosis virus A (TNV-A) to be replicated. STNV-1 has a single-stranded RNA (ssRNA) genome that is 1,239 nucleotides (nt) in length and has a single long open reading frame (ORF) that encodes the capsid protein (CP) of 21.6 kDa. The CP ORF is flanked by 5′ and 3′ untranslated regions (UTRs) of 29 and 622 nt, respectively (1). STNV-1 was the first satellite virus to be discovered (2); its helper virus, TNV-A, belongs to the genus *Alphanecrovirus*, family *Tombusviridae*, and is also a linear positive-sense, single-stranded RNA virus (https://talk.ictvonline.org/ictv-reports/ictv_online_report). TNV-A has a worldwide distribution and infects and causes necrosis in a number of hosts of economic significance, including tobacco, vegetables, and ornamental plants. Both STNV-1 and TNV-A form isometric virions of 22 to 25 nm and 28 to 35 nm in diameter comprising the STNV-1 and TNV-A CPs, respectively. The structure of the STNV-1 virions has been determined via X-ray diffraction, revealing a T=1 icosahedral capsid containing 60 copies of the STNV-1 CP (3, 4).

**FIG 1:**
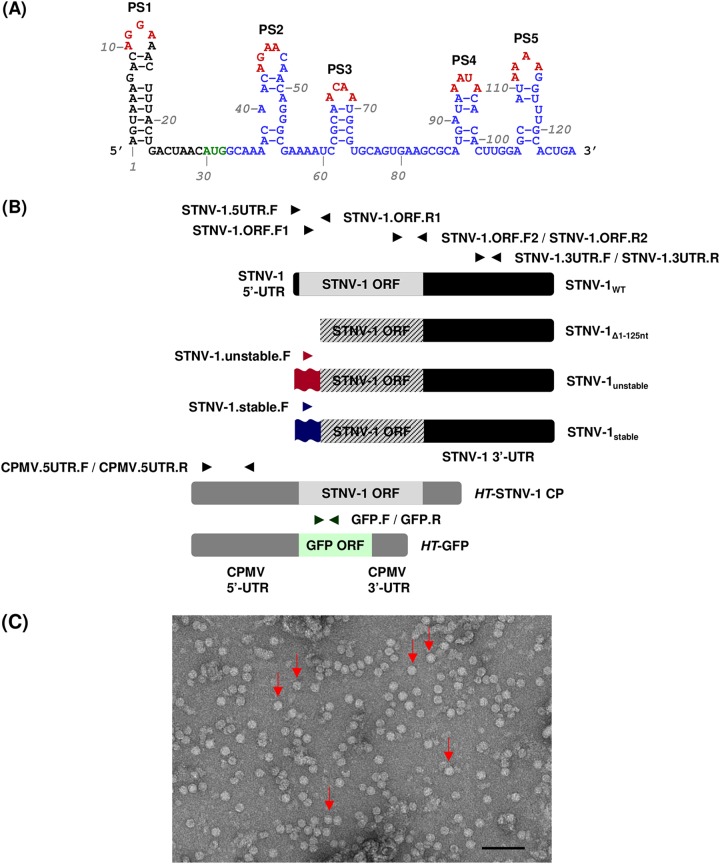
(A) Sequence and putative secondary structure of the 5′ genomic fragment of STNV-1 encompassing five putative packaging signals (PS1 to -5). Green nucleotides indicate the CP start codon, blue nucleotides indicate the CP ORF, and red nucleotides indicate the potential coat protein recognition motif (AxxA). (Adapted from reference 14.) (B) The STNV-1_WT_ genome consists of one ssRNA molecule containing one long ORF (light gray box) flanked by 5′- and 3′-UTRs (black boxes). The STNV-1_Δ1–125_ construct lacks nucleotides 1 to 125 from the 5′ terminus, which overlaps with the beginning of the CP ORF, so this construct cannot direct the expression of CP, as indicated by the black diagonal lines. The STNV-1_unstable_ and STNV-1_stable_ constructs have modified 5′ termini indicated as red and blue double waves, respectively, and these also render the ORF nonfunctional, as indicated by the diagonal lines. The *HT*-STNV-1 CP and *HT*-GFP constructs contain the STNV-1 CP ORF (light gray box) and GFP ORF (light green box) flanked by the 5′- and 3′-UTRs of CPMV (dark gray boxes). The names of oligonucleotide primers used for qPCR analysis and their positions relative to the constructs are indicated by arrows. Detailed information on the ability of each oligonucleotide primer pair to detect each construct can be provided upon request. (C) Particles produced by the pEAQ-*HT*-STNV-1 CP construct as visualized by TEM. A selection of the visible particles are indicated by red arrows and the scale bar is 100 nm.

STNV-1 is one of the smallest known viral structures and is therefore a tractable model system to study genome packaging of RNA viruses. In general, viruses encapsidate their own genome in preference to other nucleic acids. In several cases, specific sequences and/or structures that act as packaging signals (PSs) have been identified in viral RNAs. Removal of the PSs adversely affects RNA packaging *in vivo*, and their addition to unrelated RNAs can lead to their encapsidation (see, e.g., references 5 to 9). In the case of STNV-1, no such well-defined PS has been identified, and no obvious sequence specificity in the interactions between the STNV-1 CP and its RNA has been reported. In fact, structure-based assembly studies indicate that non-sequence-specific RNA-protein interactions across the length of the STNV-1 genome drive the process, including electrostatic interactions between the negatively charged nucleic acid and the positively charged N terminus of the CP (10–13). Recently, a degenerate RNA-binding motif that consists of a short stem-loop structure with a loop sequence of AxxA has been implicated in viral RNA-CP interactions *in vitro* using systematic evolution of ligands by exponential enrichment (SELEX) (11). The interaction appears to be sequence specific and present in the context of variable secondary structures (13). This motif is present in multiple copies in the STNV-1 genome, and five of these putative PSs in the 5′-terminal 127-nt-long fragment of the genome were shown to collectively promote successful encapsidation by the STNV-1 CP *in vitro* (14). Site-directed mutagenesis of all the putative PSs in the 5′ region of the STNV-1 genome supported the notion that they are crucial for assembly *in vitro* and led to the construction of two versions of the STNV-1 genome modified at their 5′ termini and considered “unstable” and “stable” with regard to their secondary structures and hence encapsidation potential (15) (Fig. 1A). In STNV-1_unstable_, the modified central PS, PS3, is unlikely to fold spontaneously to form the required stem-loop, and this construct did not support complete virus-like particle (VLP) assembly *in vitro*; conversely, PS3 is more stable in STNV-1_stable_, and this was shown to be a better assembly substrate than wild-type STNV-1 (STNV-1_WT_) *in vitro* (15). Similar *in vitro* studies have been performed with other viruses lacking well-defined PSs, including bacteriophages (16) and human viruses such as hepatitis B virus (17) and hepatitis C virus (18).

Since many single-stranded RNA viruses are important pathogens, identifying their PSs opens new possibilities for novel approaches to antiviral drug design and therapy, including, for example, the use of aptamers that mimic or inhibit encapsidation of the viral genome. In light of the above, the aim of the present study was to assess the significance of the putative PSs of the STNV-1 genome for RNA packaging *in planta*. To this end, we used agrobacterium-mediated transient expression (agroinfiltration) to introduce STNV-1 RNA variants into Nicotiana benthamiana in the presence of an excess supply of the STNV-1 CP and used reverse transcription followed by quantitative PCR (RT-qPCR) and next generation sequencing (NGS) to investigate the specificity of encapsidation of virus- and host-derived RNAs. In addition, plants were inoculated with STNV-1 in the presence of the helper TNV-A, and the effect of the presence of mutant STNV-1 RNA on encapsidation was examined. Overall, our results indicate that the presence or absence of the previously identified PSs did not have any discernible effect on the encapsidation efficiency of the viral RNAs. However, the preferential packaging of certain host RNAs suggests that the process is not random or dependent solely on RNA abundance within the cells.

## RESULTS

### NGS analysis of host and infiltrated RNAs encapsidated by STNV-1 CP following transient expression.

To investigate the role of putative packaging signals in STNV-1 RNA encapsidation within plants, it was necessary to develop a system for the consistent expression of the CP. Although full-length STNV-1 RNA serves as an mRNA for CP during infection, the previously identified PSs lie near the 5′-UTR and overlap the N terminus of the CP-coding region. Thus, any mutations in this region not only would affect any RNA structure but also would greatly influence CP expression and function. To provide a consistent source of CP, the gene encoding it was inserted into pEAQ-*HT* (19) to give plasmid pEAQ-*HT*-STNV-1 CP (Fig. 1B), which produces high levels of STNV-1 CP as a result of positioning the CP sequence between the modified cowpea mosaic virus (CPMV) RNA-2 “hypertranslatable” UTRs (19, 20). When infiltrated either on its own or together with pEAQ-STNV-1_WT_ (containing the complete STNV-1 RNA sequence under the control of the 35S promoter), large quantities of typical STNV-1 particles could be purified from infiltrated tissue (Fig. 1C). In contrast, few, if any, particles could be detected in tissue infiltrated with pEAQ-STNV-1_WT_ alone, suggesting that the expression of CP from pEAQ-STNV-1_WT_ is not sufficient to produce detectable VLPs using the production scale and extraction techniques employed here.

**FIG 2:**
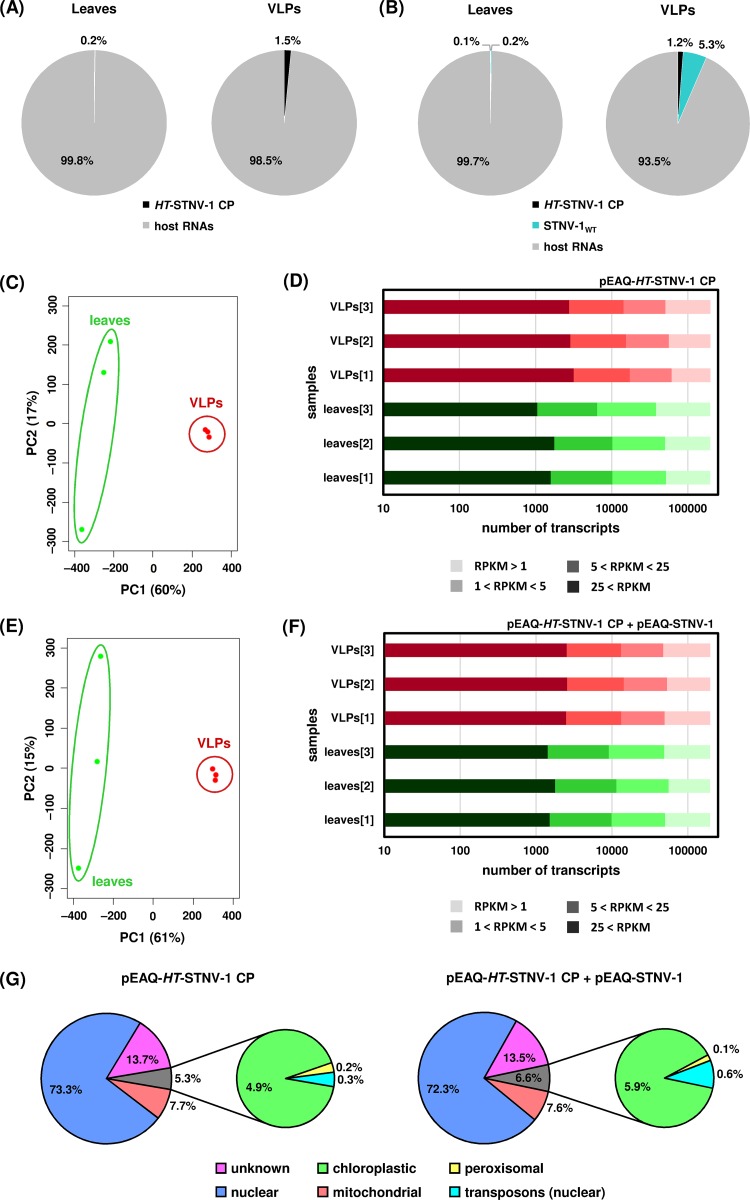
(A) Percentages of reads mapping on *HT*-STNV-1 CP and host transcripts in the leaves and in the virions purified from agroinfiltrated leaves. (B) Percentages of reads mapping on *HT*-STNV-1 CP, STNV-1_WT_, and host transcripts in the leaves and in the virions purified from agroinfiltrated leaves. (C and E) Principal-component analysis of host transcript levels in the leaves (green dots) and in the virions (red dots) following agroinfiltration with pEAQ-HT-STNV-1 CP alone (C) and pEAQ-HT-STNV-1 CP together with pEAQ-STNV-1_WT_ (E). (D and F) Numbers of N. benthamiana transcripts not expressed (RPKM < 1), expressed at low levels (1 < RPKM < 5), expressed at medium levels (5 < RPKM < 25), and expressed at high levels (25 < RPKM) in each NGS sample. (G) Percentages of transcripts and reads derived from distinct species of encapsidated host RNAs.

To examine the ability of the transiently expressed VLPs to encapsidate specific RNA molecules, N. benthamiana leaves agroinfiltrated with pEAQ-*HT*-STNV-1 CP alone and pEAQ-*HT*-STNV-1 CP together with pEAQ-STNV-1_WT_ were collected at 7 days postagroinfiltration, and total leaf RNA and RNA within purified VLPs were analyzed using RNA sequencing. Both the *HT*-STNV-1 CP and STNV-1_WT_ RNAs (produced from pEAQ-*HT*-STNV-1 CP and pEAQ-STNV-1_WT_, respectively) were enriched in the VLPs compared to the leaves, the latter more than the former. Specifically, in N. benthamiana leaves agroinfiltrated with pEAQ-*HT*-STNV-1 CP alone, the VLP-to-leaf ratio of the *HT*-STNV-1 CP RNA, based on reads per kilobase per million (RPKM) values, is 6.5 ± 3.5. In N. benthamiana leaves coagroinfiltrated with pEAQ-*HT*-STNV-1 CP together with pEAQ-STNV-1_WT_, the VLP-to-leaf ratios of the *HT*-STNV-1 CP and pEAQ-STNV-1_WT_ RNAs are 11.7 ± 6.4 and 21.9 ± 17.1, respectively. Although the standard deviations of the ratios are high, the enrichment of viral RNAs in the VLPs is statistically significant in all three cases (*P* < 0.05; one-tailed Student *t* test). Nevertheless, up to 50% of the total number of reads within the VLPs correspond to cytoplasmic rRNA (see Table S1 in the supplemental material), and the vast majority of protein-coding RNA (>90%) was of host origin (Fig. 2A and B), a situation similar to that reported previously for cucumber necrosis virus (CNV) (21).

Principal-component analysis (PCA) of the host transcript levels showed that the analyzed samples formed distinct groups based on the origin of the RNA, from either leaf tissue or the VLPs, and this constituted the major source of variation (ca. 60%) (Fig. 2C and E) among the data sets. On average, over 45,000 N. benthamiana transcripts were found to be expressed at detectable levels (RPKM > 1) in the leaves, and ca. 20% of these are considered highly (RPKM > 25) or moderately (5 < RPKM < 25) expressed (Fig. 2D and F). The number of highly or moderately expressed transcripts in the VLPs is even higher, close to 30% (Fig. 2D and F). The overall profiles of the packaged transcripts are very similar following agroinfiltration with pEAQ-*HT*-STNV-1 CP alone (see Data Set S1 in the supplemental material) or pEAQ-*HT*-STNV-1 CP together with pEAQ-STNV-1_WT_ (see Data Set S2 in the supplemental material), and their predicted origin is shown in Fig. 2G.

**FIG 3:**
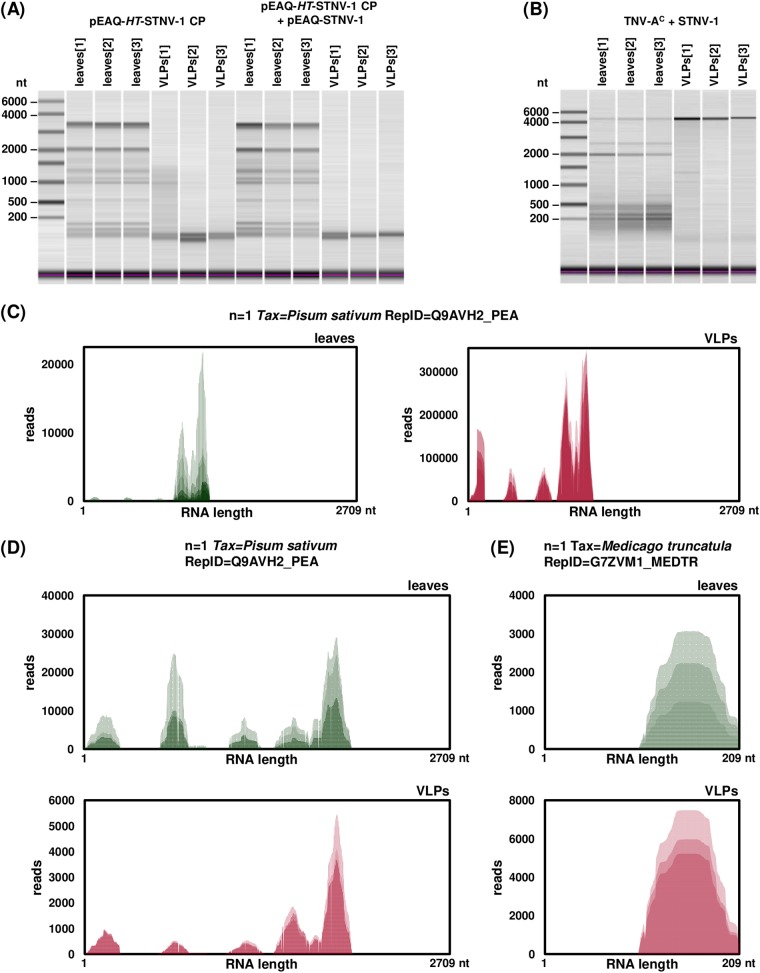
(A and B) Capillary electrophoresis of total RNA extracted from leaves and VLPs following agroinfiltration (A) and infection (B). (C to E) Visualization of reads mapped to the most abundantly encapsidated N. benthamiana transcript following agroinfiltration (C) and the two most abundantly encapsidated N. benthamiana transcripts following infection (D and E). Each NGS biological replicate is represented by a different shade of green (leaves) or red (VLPs).

The two most abundant transcripts in the VLPs (see Table S2 in the supplemental material) are a putative senescence-associated protein with 50% identity to the UniProtKB cluster “regulator of rDNA transcription protein 15” and an rRNA intron-encoded homing endonuclease. Since capillary electrophoresis of RNA extracted from the VLPs prior to the library construction did not reveal any major RNA species present (Fig. 3A), the possibility that fragments rather than intact RNA molecules are encapsidated was investigated by visualizing the alignment of the reads to the most abundant transcript (Fig. 3C). The distribution of the reads is not uniform over the length of the transcript, and specific areas are overrepresented; however, the distribution of the reads in the leaf-derived transcript is very similar, suggesting that this is most likely a reflection of bias during PCR amplification of the library rather than selective encapsidation of specific segments. However, it is unclear whether the cellular and thus the encapsidated RNA is intact or fragmented.

Differential expression analysis using DESeq2 revealed 729 and 737 N. benthamiana transcripts present in the VLPs at a statistically significantly increased amount compared to that in leaf tissue following agroinfiltration with pEAQ-HT-STNV-1 CP alone or pEAQ-HT-STNV-1 CP plus pEAQ-STNV-1_WT_, respectively, with 506 transcripts common for both data sets. The fold change of the RNA levels in the virions compared to those in the leaf tissue ranges from 2 to over 100, and the adjusted *P* values are lower than 0.05 as calculated by DESeq2.

### RT-qPCR analysis of infiltrated RNAs encapsidated by STNV-1 CP following transient expression.

**FIG 4:**
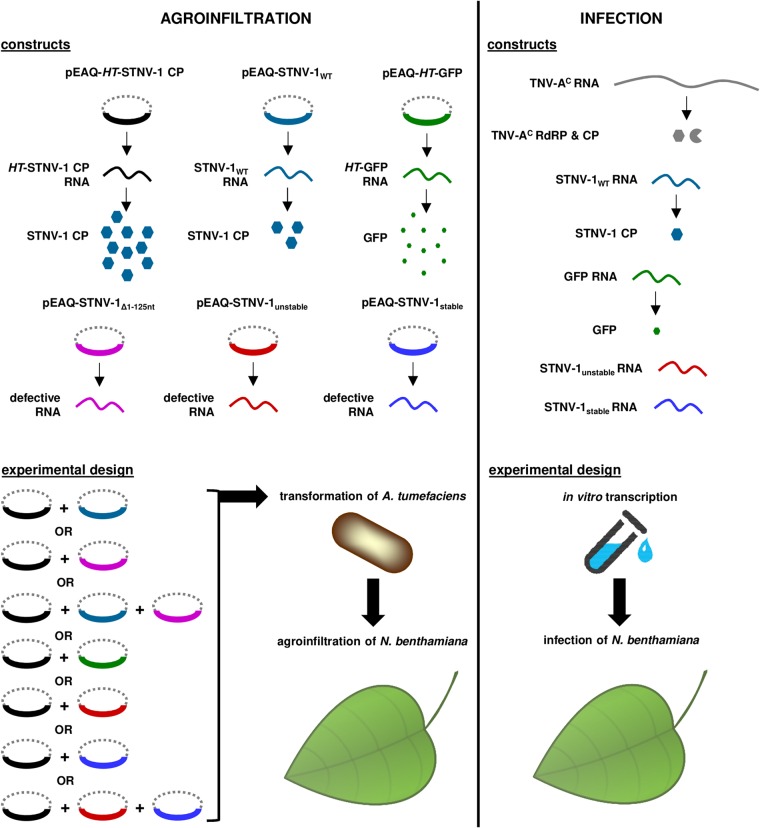
Schematic representation of the constructs used for agroinfiltration and infection together with the experimental design for each procedure.

**FIG 5:**
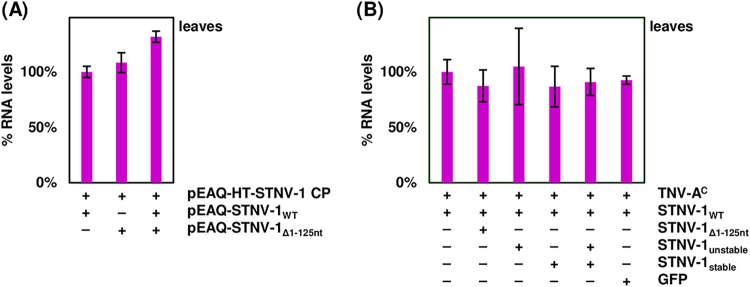
(A) RT-qPCR analysis of total viral RNAs (*HT*-STNV-1 CP, STNV-1_WT_, and STNV-1_Δ1-125_) in the leaves of plants agroinfiltrated with pEAQ-*HT*-STNV-1 CP in addition to pEAQ-STNV-1_WT_, STNV-1_Δ1–125_, or both, normalized to N. benthamiana 18S rRNA. (B) RT-qPCR analysis of STNV-1_WT_ RNA in the leaves of plants infected with STNV-1 _WT_ and TNV-A^C^ in addition to STNV-1_unstable_, STNV-1_stable_, or both, normalized to N. benthamiana 18S rRNA. Samples were collected at 7 days postagroinfiltration or postinfection of N. benthamiana plants. At least three independent repetitions were performed in triplicate, and error bars represent standard deviation. The presence or absence of nonreplicating RNAs can be found in Table 1. At least three independent repetitions were performed in triplicate, and error bars represent standard deviation.

**FIG 6:**
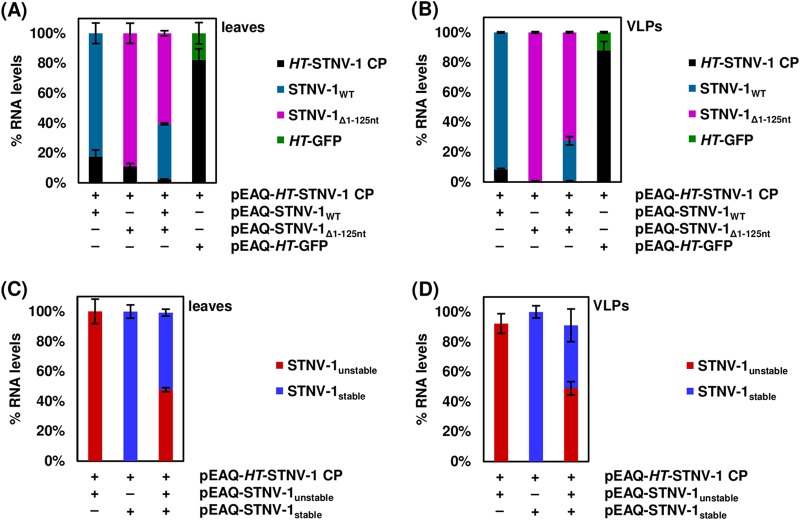
(A) RT-qPCR analysis of viral RNAs in the leaves of plants agroinfiltrated with pEAQ-*HT*-STNV-1 CP in addition to pEAQ-STNV-1_WT_, STNV-1_Δ1–125_, both aforementioned constructs, or pEAQ-*HT*-GFP. (B) RT-qPCR analysis of viral RNAs in the VLPs after purification from the same plants. (C) RT-qPCR analysis of viral RNAs in the leaves of plants agroinfiltrated with pEAQ-*HT*-STNV-1 CP in addition to pEAQ-STNV-1_unstable_, STNV-1_stable_, or both. (D) RT-qPCR analysis of viral RNAs in the VLPs after purification from the same plants. All samples were collected at 7 days postagroinfiltration of N. benthamiana plants. At least three independent repetitions were performed in triplicate, and error bars represent standard deviation.

Based on the RNA sequencing results showing preferential encapsidation of viral RNAs, the pEAQ transient-expression system (19) was then used to produce *in planta* wild-type STNV-1 RNA, STNV-1_Δ1–125_ RNA (which lacks nucleotides 1 to 125 and therefore all five previously identified putative PSs [14] as well as the ability to direct the expression of the STNV-1 CP [since the beginning of the CP ORF overlaps with these 5′ 125 nucleotides]), and two synthetic versions of the STNV-1 genome with modifications to the 5′ 125 nucleotides that are expected to form significantly more “unstable” and “stable” forms of the putative packaging signal than the wild-type sequence (15) (Fig. 1) (sequences are available upon request). These are the same sequences used to provide evidence of the effect of the PSs on STNV-1 packaging *in vitro* (15); because of the overlap between these PSs and the CP ORF, these constructs also lack the ability to direct the expression of the CP. These constructs were introduced into N. benthamiana plants separately or in combination via agroinfiltration in the presence of pEAQ-*HT*-STNV-1 CP (Fig. 4) to provide a consistent level of CP. Following RNA extraction from leaves and purification of VLPs, a collection of oligonucleotide primers was used to quantify each RNA species in RT-qPCR assays with comparable efficiency above 95% (data available upon request). Total viral RNA levels in the leaves were similar (Fig. 5A) as quantified with primer pair STNV-1.ORF.F2/STNV-1.ORF.R2, which binds toward the end of the STNV-1 CP ORF and amplifies all constructs (Fig. 1A), suggesting that there is a maximum quantity of RNA that can accumulate in the cell regardless of the number of different constructs introduced; however, the relative abundance of specific RNAs differed (Fig. 6A), reflecting variation in the accumulation of the RNAs from the pEAQ vectors. The STNV-1_Δ1–125_ RNA was consistently the most abundant, followed by STNV-1_WT_ and *HT*-STNV-1 CP (Fig. 6A), while STNV-1_unstable_ and STNV-1_stable_ were present in equal quantities (Fig. 6C). Preferential encapsidation was not detected between STNV-1_WT_ and STNV-1_Δ1–125_ or between STNV-1_unstable_ and STNV-1_stable_, either when the constructs were supplied separately or in competition experiments; conversely, the amount of RNA present in the leaf cells (Fig. 6A and C) appears to be the major determinant of encapsidation efficiency (Fig. 6B and D). This is particularly clear in the case of STNV-1_unstable_ and STNV-1_stable_, which when present in a 1:1 ratio in the cell are encapsidated in the same ratio (Fig. 6C and D), representing a striking difference from the aforementioned *in vitro* work on these same RNAs (15). A pEAQ-*HT* vector producing high levels of green fluorescent protein (GFP) confirmed that the observations are also applicable to nonviral RNAs (Fig. 6A and B), illustrating that an unrelated RNA can be encapsidated by STNV-1 CP at high efficiency, comparable to that for STNV-1_WT_, despite this RNA being unlikely to contain STNV-1-specific PSs. Finally, no purified VLPs could be visualized by transmission electron microscopy (TEM), and no encapsidated RNAs could be amplified in samples lacking pEAQ-*HT*-STNV-1 CP. Overall, neither the complete removal of the five PSs from the viral genome (STNV-1_Δ1–125_) nor their modification (STNV-1_unstable_ and STNV-1_stable_) resulted in loss or alteration of relative encapsidation efficiency.

### RT-qPCR analysis of encapsidated RNAs following infection.

**FIG 7:**
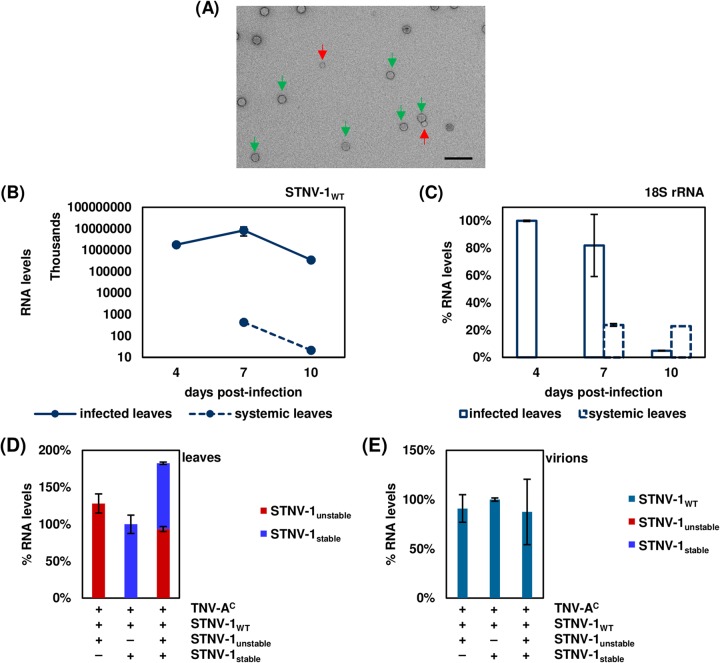
(A) Virions produced by STNV-1_WT_ and its helper virus TNV-A^C^ from a mechanical inoculation as visualized by TEM. The purification method copurifies larger TNV-A^C^ particles (indicated by green arrows) with the smaller STNV-1 particles (indicated by red arrows). The scale bar is 100 nm. (B and C) Time course of STNV-1 titers (B) and N. benthamiana 18S rRNA (C) in infected and systemic N. benthamiana leaves at 4, 7, and 10 days postinfection as determined by RT-qPCR analysis. (D) RT-qPCR analysis of viral RNAs in the leaves of plants infected with STNV-1_WT_ and TNV-A^C^ in addition to STNV-1_unstable_, STNV-1_stable_, or both. Only STNV-1_unstable_ and STNV-1_stable_ are shown here; STNV-1_WT_ levels are orders of magnitude higher and are shown in Fig. 5B. (E) RT-qPCR analysis of viral RNAs in the virions after purification from the same plants. All samples were collected at 7 days postinfection of N. benthamiana plants. At least three independent repetitions were performed in triplicate, and error bars represent standard deviation.

To compare the encapsidation specificity assessed via transient expression with that in the context of a viral infection, N. benthamiana leaves were mechanically inoculated with *in vitro*-transcribed RNAs corresponding to STNV-1_WT_ and its helper virus TNV-A^C^, a combination that causes systemic infection in N. benthamiana (22), together with STNV-1_unstable_, STNV-1_stable_, or STNV-1_unstable_ and STNV-1_stable_ combined or with GFP (Fig. 4). Initial experiments revealed that particles of both TNV-A^C^ and STNV-1 could be visualized in infected leaves (Fig. 7A), and the viral RNA titer was highest at 7 days postinfection (Fig. 7B) in both inoculated and systemically infected leaves. Inoculated leaf tissue was almost completely necrotic at 10 days postinfection as reflected by the N. benthamiana 18S rRNA levels (Fig. 7C). The mutants STNV-1_unstable_ and STNV-1_stable_, which carry modifications in the 5′ terminus, and the GFP RNA used as a control were detectable in the inoculated leaves at 7 days postinfection at approximately 0.01% of STNV-1_WT_ levels (Fig. 5B), consistent with their inability to be replicated by the helper TNV-A^C^ RNA-dependent RNA polymerase (RdRP). The presence of these RNAs within the cells was not dependent on the presence of STNV-1_WT_ and/or TNV-A^C^ (Table 1), ruling out the possibility that the RNAs might be protected from degradation by encapsidation by either the STNV-1 or TNV-A^C^ CP. STNV-1_WT_ RNA has been reported to survive in the absence of its helper virus for at least 10 days postinoculation (23) due to extensive secondary structure that increases its stability (24). Similarly, the STNV-1_unstable_, STNV-1_stable_, and GFP RNAs appeared to be able to survive, possibly assisted by the use of bentonite, which is known to protect RNA from degradation (25), during the inoculation process. Consistent with the agroinfiltration experiments, STNV-1_unstable_ and STNV-1_stable were_ present in equal amounts in the cells following coinfection (Fig. 7D) but were below the limits of detection in preparations of particles (Fig. 7A), which appeared to contain solely STNV-1_WT_ even when leaves were coinoculated with all three constructs (Fig. 7E).

**TABLE 1:** Detection of RNA in leaves by RT-qPCR at 7 days postinfection

Expt	Inoculum^*a*^					Leaves^*b*^			
	TNV-A^C^	STNV-1_WT_	STNV-1_unstable_	STNV-1_stable_	GFP	STNV-1_WT_	STNV-1_unstable_	STNV-1_stable_	GFP
1	Y	Y	N	N	N	++	−	−	−
2	Y	Y	Y	N	N	++	+	−	−
3	Y	Y	N	Y	N	++	−	+	−
4	Y	Y	Y	Y	N	++	+	+	−
5	Y	Y	N	N	Y	++	−	−	+
6	Y	N	N	N	N	−	−	−	−
7	Y	N	Y	N	N	−	+	−	−
8	Y	N	N	Y	N	−	−	+	−
9	Y	N	Y	Y	N	−	+	+	−
10	Y	N	N	N	Y	−	−	−	+
11	N	Y	N	N	N	+	−	−	−
12	N	Y	Y	N	N	+	+	−	−
13	N	Y	N	Y	N	+	−	+	−
14	N	Y	Y	Y	N	+	+	+	−
15	N	Y	N	N	Y	+	−	−	+
16	N	N	N	N	N	−	−	−	−
17	N	N	Y	N	N	−	+	−	−
18	N	N	N	Y	N	−	−	+	−
19	N	N	Y	Y	N	−	+	+	−
20	N	N	N	N	Y	−	−	−	+

a Y, the RNA was used for infection; N, the RNA was not used for infection.

b ++, the RNA was detected at high levels; +, the RNA was detected at low levels; −, the RNA was not detected.

### NGS analysis of encapsidated host and viral RNAs following infection.

**FIG 8:**
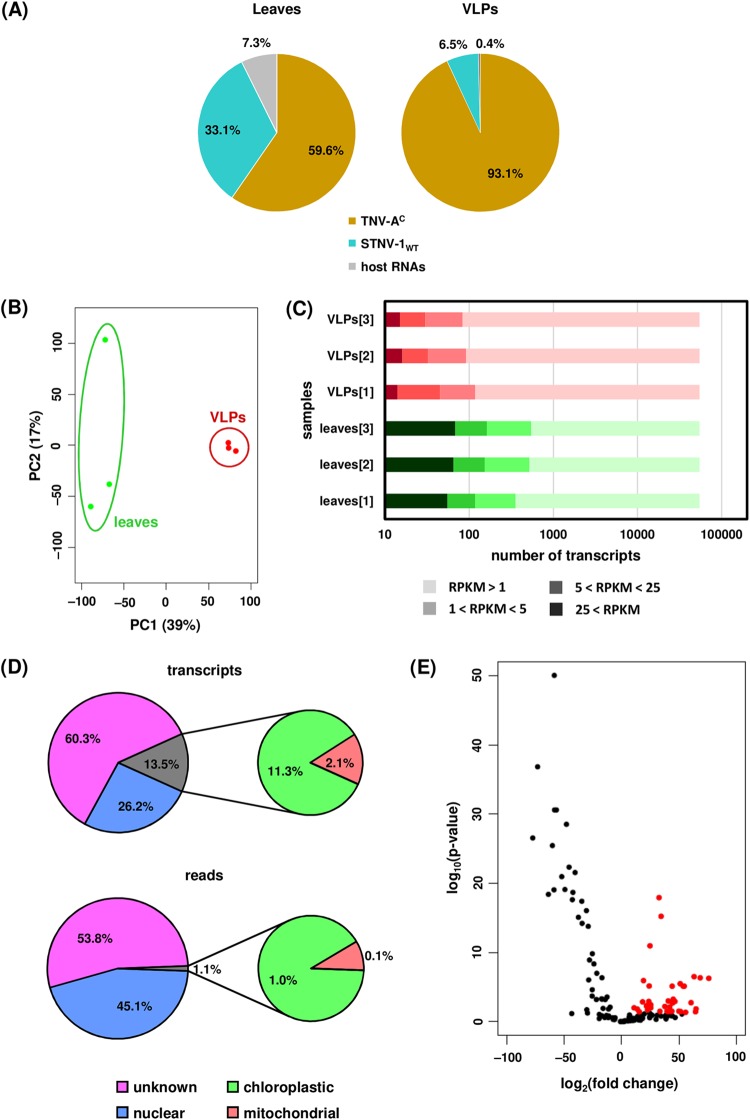
(A) Percentages of reads mapping on TNV-A^C^, STNV-1_WT_, and host transcripts in the leaves and in the virions purified from infected leaves. (B) Principal-component analysis of host transcript levels in the leaves (green dots) and in the virions (red dots). (C) Numbers of N. benthamiana transcripts not expressed (RPKM < 1), expressed at low levels (1 < RPKM < 5), expressed at medium levels (5 < RPKM < 25), and expressed at high levels (25 < RPKM) in each NGS sample. (D) Percentages of transcripts and reads derived from distinct species of encapsidated host RNAs. (E) Volcano plot following differential expression analysis; red dots signify host transcripts present in at least a 2-fold-increased quantity in the purified virions compared to leaf tissue (adjusted *P* value, <0.05).

The possible incorporation of host-derived RNAs into virions during infection was examined following inoculation of N. benthamiana leaves with STNV-1_WT_ and its helper virus, TNV-A^C^. The inoculated leaves were collected at 7 days postinfection, and both total leaf RNA and RNA within purified virions was analyzed using RNA sequencing (see Data Set S3 in the supplemental material). In contrast to the agroinfiltration experiments, the percentage of reads derived from rRNA was very low (<0.5%; see Table S3 in the supplemental material) and the vast majority of protein-coding RNA was of viral origin, with only 7.3% and 0.4% of the reads derived from host protein-coding RNAs in the leaf tissue and the virions, respectively (Fig. 8A). The percentage of encapsidated host RNAs is in agreement with the reports that less than 1% of the RNA in CNV (21) and brome mosaic virus (BMV) (26) virions originates from the plant host. However, it is important to note that because TNV virions could not be separated from STNV-1 particles, these results relate to encapsidation within TNV particles more than they do to STNV-1 particles. This likely explains the apparent discrepancy with results obtained from agroinfiltration of STNV-1-based constructs.

Principal-component analysis (PCA) of the host transcript levels showed that the analyzed samples formed distinct groups based on the origin of the RNA, from either the leaf tissue or the virions, and this constituted the major source of variation (ca. 70%) (Fig. 8C) among the data sets. In total, 141 host transcripts were identified as present (RPKM > 1) within the virions in at least one of the three replicates processed. Fifty-eight of these are present solely following infection, while the rest can be detected also following agroinfiltration. Their predicted origin is shown in Fig. 8D, with approximately 1% of the encapsidated host RNAs being derived from organelle transcripts. This is in contrast to previous observations that chloroplastic mRNAs are the most abundantly encapsidated by CNV (21), a result attributable to the fact that a small proportion of the CNV CP is localized in the chloroplasts (27), while there are no such reports for the STNV-1 CP.

The two most abundant transcripts in the virions account for over 50% of the host encapsidated RNA (see Table S4 in the supplemental material). One of them encodes the same putative senescence-associated protein that is most abundant in the VLPs following agroinfiltration, while the other is a putative uncharacterized protein now considered obsolete in UniProtKB. Interestingly, almost half of the most abundant transcripts in the virions are the same as those in VLPs following agroinfiltration (Table S4). Visualization of the distribution of the reads on these transcripts showed that parts of the sequence are overrepresented in both the leaves and the virions (Fig. 3D and E), in agreement with the previous observations for the agroinfiltration experiments and reinforcing the notion that encapsidation of RNA species by STNV-1 CP is driven predominantly by their abundance in the cell.

**FIG 9:**
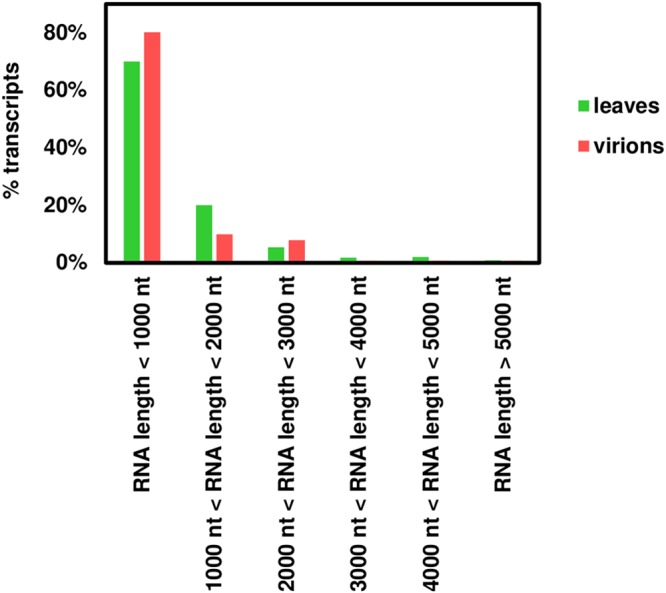
Length distribution of transcripts present in the leaves and in the virions following infection.

Differential expression analysis using DESeq2 revealed 40 N. benthamiana transcripts present in the virions at a statistically significantly increased amount compared to those in leaf tissue (Fig. 8E). The fold change of the RNA levels in the virions compared to those in the leaf tissue ranges from 2 to over 100, and the adjusted *P* values are lower than 0.05 as calculated by DESeq2. The most efficiently encapsidated transcripts are listed in Table S5 in the supplemental material. The imperfect correlation between transcript abundances in the leaf and in the virions suggests that specific RNAs may have higher affinity for the CP; however there is no correlation (as illustrated by Pearson’s correlation coefficient) between this preference as quantified by the fold change of the RNA levels in the virions compared to those in the leaf tissue and the number of AxxA motifs, either absolute or normalized to transcript length, found in the sequence. For instance, the transcript encoding the putative uncharacterized protein designated n=1 Tax=Glycine max RepID=I1L3I4_SOYBN (Table S5) has fewer than 20 AxxA motifs in its sequence and is efficiently encapsidated (almost a 10-fold change); conversely, the transcript encoding the putative uncharacterized protein designated n=1 Tax=Vitis vinifera RepID=A5CBG1_VITVI has over 200 AxxA motifs in its sequence and is underrepresented within the virions (0.24-fold change). It is conceivable that some of these motifs may not be found in an optimal structural context to act as PSs and promote encapsidation. Notably, the vast majority (90%) of host transcripts within the virions are less than 2,000 nt in length, and only three are longer than 3,000 nt. Even though this may be due space constraints, the length distribution of the transcripts in the virions is not significantly different from that of the transcripts in the leaves (Fig. 9).

## DISCUSSION

In this study, two different methods (transient expression and viral infection) were used to assess the encapsidation competence of specific RNAs of interest: viral RNAs, mutants that are expected to differ in their capacity to be packaged, and nonviral RNAs. Moreover, NGS was used to paint a broad picture of what is packaged inside STNV-1 particles *in vivo*. The results reveal that the presence of the PSs identified in the STNV-1 genome *in vitro* is insufficient to account for the selectivity of packaging of STNV-1 RNA observed during infection. The data presented here indicate that packaging of RNA, whether of viral or host origin, is dictated primarily by abundance within the cell, suggesting that there may not be specific sequence-based packaging signals directing the packaging of RNA into STNV-1 capsids. Indeed, RNA molecules specifically designed to differ from the wild-type genome in their capacity to be packaged (STNV-1_Δ1–125_, STNV-1_stable_, and STNV-1_unstable_) were packaged largely in proportion to their abundance in the cell during transient expression, even when in direct competition with one another or with STNV-1_WT_.

While the presence of additional AxxA motifs throughout the STNV-1 genome (15) might compensate for the mutations at the 5′ termini of these RNAs, this would not explain the relative efficiency of packaging for GFP-coding RNA, which has no relation to viral RNA. It is also possible that the expression system used in the transient-expression experiments led to the expression of excess coat protein which may have reduced specificity of packaging. However, if this were the case, the specificity of the putative PSs would have to be very weak indeed to be completely undetected in direct competition experiments, which *in vitro* work indicates should show a clear packaging preference. In any case, these *in vivo* results clearly differ from those obtained from *in vitro* experiments (15).

It has previously been suggested that the CP N terminus may be responsible for nonspecific interactions with the viral RNA (28), and, the putative PSs notwithstanding, assembly was previously found to be heavily influenced by electrostatic interactions (13). Alternatively, the packaging phenomena observed *in vitro* may depend on predefined quantities and ratios of viral RNA and CP that would allow their detection and do not accurately reflect the conditions within the cell where the viral RNAs must be selected from a complex mixture of cellular RNAs. The link between transcript abundance in the cell and in viral particles is not unique to STNV-1: it has been shown that in at least two retroviruses (murine leukemia virus and human immunodeficiency virus [HIV] type 1), packaging of RNA is determined largely by abundance within the cell, in a manner that is similar to the situation described here with STNV-1 (29). In the cases of these retroviruses, it was found that host RNA essentially compensates for viral RNA during particle formation in the absence of viral replication. Patel et al. (15) suggested it is possible that the *in vitro*-identified STNV-1 PSs may be able to interact with CP only when they are present on nascent RNA molecules synthesized as a result of replication; however, we show that any potential role *in vivo* is overshadowed by the sheer amount of STNV-1 RNA produced in infected cells as a consequence of replication, since over 90% of the cellular protein-coding RNA is viral, with approximately one-third derived from STNV-1 and the rest from TNV-A^C^. Indeed, the local concentration is likely to be even higher in replication complexes.

The imperfect correlation between transcript abundance in the cell and in the viral particles, particularly for host RNA, suggests that there may well be sequences which are more amenable to packaging by STNV-1 CP than others. However, the data presented here do not point to any obvious simple sequence, and they certainly weigh against AxxA motifs as true packaging signals *in vivo*. What seems more likely is that certain more complex features of RNA have an impact on encapsidation competence, without a specific sequence being consistently involved. Such features are likely to involve RNA length or structure, insofar as RNA must be capable of compacting enough to be packaged within a viral particle. Alternatively, or perhaps in addition, 3′-UTR length may be involved; indeed, Comas-Garcia et al. (30) found that the length of the 3′-UTR of a transcript plays a significant role in selectivity of packaging in HIV, possibly due to that region being devoid of ribosomes.

All of this does not mean that AxxA PSs have no relevance or utility. Potential uses for PS-containing RNAs might include the packaging of desired RNA molecules in protein containers *in vitro*, since simple motifs do seem to contribute to packaging efficiency in a carefully controlled *in vitro* context (15). However, the use of such PS-containing molecules as decoys for antiviral purposes *in vivo* seems, at present, to be unlikely. Indeed, RNAs with modified PSs could not be used as decoys to block or slow down STNV-1 infection, since they do not replicate independently and therefore are present in very low quantities in the leaves compared to STNV-1_WT_ and below the detection limit in the virus particles. It should be noted that the absence of any clear-cut PSs responsible for packaging on a viral RNA is not restricted to STNV-1. For example, recent work on CPMV, a member of the order *Picornavirales*, has shown that incorporation of the two genomic RNAs into particles is driven not by the presence of specific sequences but rather by the ability of the RNAs to replicate (31). Similarly, flock house virus (FHV) genome packaging requires coat protein translated from replicating viral RNA (32). Thus, the idea that PSs must be present on viral RNAs to ensure their selective packaging does not appear to be generally applicable.

Another potentially significant finding from the NGS analysis is the packaging of transposons within STNV-1 particles. Transposons have been previously found to be encapsidated by both plant (21, 26) and animal (33) viruses. In this study, transposable elements and retrotransposons were present in the VLPs at very low levels, in agreement with previous studies (21), and include members of the Gypsy family, such as Tf2 and Ty3, and the Copia family, such as TNT 1 to 94, all belonging to class I long terminal repeat (LTR) transposable elements. Additionally, RNAs encoding two putative uncharacterized proteins designated n=2 Tax=Vitis vinifera RepID=A5B1A3_VITVI and n=1 Tax=Vitis vinifera RepID=A5BDH9_VITVI and shown by Ghoshal et al. (21) to contain conserved sequences corresponding to the various proteins found in retrotransposons were also present in VLPs. Packaging of host RNAs, especially transposons and retrotransposable elements, as reported previously for other animal and plant viruses (21, 26, 33–35), may be a universal feature that allows horizontal gene transfer and contributes to genome evolution.

## MATERIALS AND METHODS

### Molecular cloning and *in vitro* transcription.

Plasmids pEAQ-STNV-1_wt_, pEAQ-STNV-1_unstable_, and pEAQ-STNV-1_stable_ were constructed by subcloning the STNV-1-based sequences flanked by the 35S promoter and *nos* terminator (which were synthesized by GeneArt and inserted into a pMA plasmid) into pEAQ-*HT* (19), following restriction digestion with PacI and SgsI. The pEAQ-*HT*-STNV-1 CP vector was generated by subcloning the STNV-1 coat protein (CP) ORF (nt 30 to 620) into the pEAQ-*HT* overexpression vector (10) after restriction digestion with AgeI and XhoI. Vector pEAQ-STNV-1_Δ1-125_ was constructed by overlapping PCR using two external primers (pMA-tHB5′ and pMA-coreC3′) binding on the pMA vector backbone and two partially overlapping internal primers (STNV-1.Δ1-125.F and STNV-1.Δ1-125.R) responsible for the deletion of the 5′ 125 nucleotides of the STNV-1 genome. All genetic manipulations were carried out according to standard protocols (36), and oligonucleotide primers and maps of the plasmids are available upon request. Notably, since the pEAQ-*HT* plasmid encodes the P19 silencing suppressor, all RNAs expressed transiently are coexpressed with P19 from the same transfer DNA (T-DNA). *In vitro* transcription and subsequent removal of the DNA template were performed using the T7 RiboMAX Express large-scale RNA production system (Promega) as instructed by the manufacturer. For production of the qPCR absolute standard curves, PCR amplicons generated by oligonucleotide primers with the T7 promoter sequence (available upon request) were used as the template. For production of TNV-A^C^ and STNV-1 RNA transcripts, plasmids pMTC27 and pUC18-STNV-1 linearized with SmaI and BamHI, respectively, were used as templates.

### Agroinfiltration and infection.

Agroinfiltration was carried out according to standard protocols (37). Briefly, overnight cultures of Agrobacterium tumefaciens strain LBA4404 carrying the desired plasmids were pelleted and resuspended in MMA buffer (10 mM morpholineethanesulfonic acid [MES] buffer [pH 5.6], 10 mM magnesium chloride, 100 μM acetosyringone) to an optical density at 600 nm (OD_600_) of 0.4. For coinfiltration of multiple constructs, equal concentrations of agrobacterial suspensions were mixed for a final OD_600_ of 0.6. The suspensions were then infiltrated into the intercellular space of leaves of preflowering Nicotiana benthamiana plants using a needleless syringe. Infiltrated leaves were harvested at 6 to 7 days postinoculation (dpi). N. benthamiana plants were also infected with viral RNA transcripts. Briefly, 5 μg per leaf of each RNA in 0.25 M phosphate buffer (pH 9.0) with 1% (wt/vol) bentonite were applied using cotton buds on selected leaves after dusting them with carborundum, and the leaves were rinsed with water at 5 min postinfection. Infected leaves were collected at 4, 7, and 10 dpi, while systemically infected leaves were collected at 7 and 10 dpi. In all cases, N. benthamiana plants were grown in a glasshouse at 25°C under 16-h day/8-h night conditions.

### Viral particle purification and visualization.

Purification of viral particles obtained from agroinfiltration was performed at 6 to 7 dpi based on a method described previously (38). Specifically, the infiltrated areas of leaves were weighed and blended in a Waring blender with three volumes of extraction buffer (0.1 M sodium phosphate [pH 7.2] supplemented with Roche complete protease inhibitor cocktail tablets), and the crude lysate was filtered over a layer of Miracloth. The crude filtrate was then clarified by centrifugation at 15,000 × *g* for 20 min at 4°C. The clarified lysate was filtered using a 0.45-μm syringe filter and then loaded into 36-ml UltraClear ultracentrifuge tubes (Beckman Coulter) and underlaid with a 5-ml layer of 25% (wt/vol) sucrose and a 1-ml layer of 70% (wt/vol) sucrose, each prepared in 0.1 M sodium phosphate, pH 7.2. These double-sucrose cushions were ultracentrifuged in a Surespin 630 rotor at 166,800 × *g* for 3 h at 4°C. A needle was used to make a hole in the bottom of the tubes, and the bottom 2 ml of each tube was recovered and dialyzed against phosphate-buffered saline (PBS). Dialysates were clarified at 27,000 × *g* for 20 min at 4°C and then filtered with a 0.2-μm syringe filter and concentrated on an Amicon Ultra-15 centrifugal filter unit with a 100,000-molecular-weight cutoff. A final clarification of the concentrate was done by two consecutive centrifugations at 16,000 × *g* for 20 min each in a benchtop microcentrifuge.

Virus purification was performed after infection of N. benthamiana plants with TNV-A^C^ and STNV-1 RNA transcripts. Briefly, infected leaves were ground in 100 mM sodium acetate (pH 5.0) and 5 mM β-mercaptoethanol, kept on ice for 1 h, filtered through Miracloth (Merck Millipore), and centrifuged at 8,000 × *g*. The supernatant was adjusted to 8% (wt/vol) polyethylene glycol (molecular, weight 8,000) and stirred at 4°C overnight. Virus was pelleted by centrifugation at 8,000 × *g*, resuspended in 10 mM sodium acetate (pH 5.0), and centrifuged at 20,000 × *g*. Virus in the supernatant was then pelleted again by high-speed centrifugation (145,000 × *g* for 2.5 h in an MLA-80 rotor [Beckman Coulter]) at 4°C and then resuspended in 10 mM sodium acetate (pH 5.0). TNV-A^C^ and STNV-1 particles could not be separated efficiently, so further analyses proceeded with samples containing both.

Purified viral particles were negatively stained with 1% (wt/vol) uranyl acetate on carbon-coated 400-mesh copper grids and examined using an FEI Tecnai 20 transmission electron microscope.

### RNA extraction and RT-qPCR.

Total RNA was extracted from 100-mg samples of N. benthamiana leaf tissue using the Qiagen RNeasy plant minikit, and residual DNA was digested using DNase I (Promega). RNA extraction from purified virions was performed using phenol-chloroform, after treatment with micrococcal nuclease (New England Biolabs) to digest extraneous, nonencapsidated RNA, similarly to other studies (21, 26). The RNA quantity and purity were assessed using a NanoDrop Lite spectrophotometer (Thermo Fisher Scientific), and 1 μg of RNA was used in a reverse transcription reaction with random hexamers (Promega) and Superscript III reverse transcriptase (Thermo Fisher Scientific). The qPCR assays were performed in the OneStepPlus real-time qPCR system (Applied Biosystems) using the Power SYBR green PCR master mix (Applied Biosystems) and the relative standard curve quantitation method. All oligonucleotide primers were designed by Primer-BLAST (39) and are available upon request; all reagents were used according to the manufacturer’s instructions. Control reactions using RNA prior to reverse transcription were also included to exclude the possibility that DNA templates were still present.

### NGS and data analysis.

For next-generation sequencing (NGS), the RNA samples were examined with a capillary Shimadzu MultiNA microchip electrophoresis system prior to processing by Vertis Biotechnologie AG (Freising, Germany). Library construction and Illumina sequencing were performed, following rRNA depletion in the case of RNA extracted from leaves but not for that from VLPs. The overall quality of the sequencing data was assessed using FASTQC, and adapters and low-quality bases were clipped and trimmed, respectively, from the reads using Trimmomatic (40). The reads were mapped to the N. benthamiana transcriptome (v5 from http://sydney.edu.au/science/molecular_bioscience/sites/benthamiana/) (41), including the Nicotiana tabacum 26S and 18S cytoplasmic rRNA genes, 26S and 18S mitochondrial rRNA genes, and 23S and 16S chloroplastic rRNA genes (accession numbers AF479172, AJ236016, BA000042, and Z00044), together with the TNV-A^C^ (accession number AY546104) and STNV-1 (accession number L06057) genomes, using Bowtie 2 (42). Sequence alignment map (.sam) files were converted to binary alignment map (.bam) files, sorted, and indexed using SAMtools (43); alignments were visualized using Integrative Genomics Viewer (IGV) (44). Gene expression was quantified with Salmon (45), and transcripts were considered “expressed” if they had a reads per kilobase per million (RPKM) value higher than 1 (46, 47). Principal-component analysis (PCA) following variance-stabilizing transformation of the data and differential gene expression analysis with DESeq2 (48) was done in R/Bioconductor, custom scripts were written in Perl, and all functions were performed in Ubuntu. Retroelements were classified using the Gypsy Database (49), and searches for protein conserved domains were conducted using Pfam (50).

## Supplementary Material

Supplemental file 1

Supplemental file 2

Supplemental file 3

Supplemental file 4
